# Applying an Evidence-Based Community Organizing Approach to Strengthen HIV Prevention for Cisgender Women in US South: Protocol for a Mixed Methods Study

**DOI:** 10.2196/56293

**Published:** 2024-03-22

**Authors:** Anandi N Sheth, Dazon Dixon Diallo, Celeste Ellison, Deja L Er, Adaora Ntukogu, Kelli A Komro, Jessica M Sales

**Affiliations:** 1 Division of Infectious Diseases Department of Medicine Emory University School of Medicine Atlanta, GA United States; 2 Ponce de Leon Center Grady Health System Atlanta, GA United States; 3 SisterLove, Inc Atlanta, GA United States; 4 Department of Behavioral, Social, and Health Education Sciences Emory University Rollins School of Public Health Atlanta, GA United States; 5 Medical College of Georgia Augusta, GA United States; 6 Department of Epidemiology Emory University Rollins School of Public Health Atlanta, GA United States

**Keywords:** cisgender women, HIV prevention, community engagement, HIV, cisgender, United States, pre-exposure prophylaxis, PrEP, Ending the HIV Epidemic, awareness, community, community-based, prevention, community action team, HIV epidemic, evidence-based, participatory, community-engaged approach, community organizing approach, men who have sex with men

## Abstract

**Background:**

Most new HIV diagnoses among cisgender women in the United States occur in the South. HIV pre-exposure prophylaxis (PrEP), a cornerstone of the federal Ending the HIV Epidemic (EHE) initiative, remains underused by cisgender women who may benefit. Awareness and access to PrEP remain low among cisgender women. Moreover, improving PrEP reach among cisgender women requires effectively engaging communities in the development of appropriate and acceptable patient-centered PrEP care approaches to support uptake. In a community-clinic-academic collaboration, this protocol applies an evidence-based community organizing approach (COA) to increase PrEP awareness and reach among cisgender women in Atlanta.

**Objective:**

The aim of this study is to use and evaluate a COA for engaging community members across 4 Atlanta counties with high-priority EHE designation, to increase PrEP awareness, interest, and connection to PrEP care among cisgender women.

**Methods:**

The COA, consisting of 6 stages, will systematically develop the skills of community members to become leaders and advocates for HIV prevention inclusive of PrEP for cisgender women in their communities. We will use the evidence-based COA to develop and implement a PrEP-specific action plan to create broader community change by raising awareness and interest in PrEP, reducing stigma associated with HIV or PrEP, and connecting women to sexual health clinics providing PrEP services. In the first 4 stages, to prepare for and develop action plans, we will gather data from one-on-one interviews with up to 100 individuals across Atlanta to capture attitudes, motivations, and influences related to women’s sexual health with a focus on HIV prevention and PrEP. Informed by the community interviews, we will revise a sexual health curriculum inclusive of PrEP and community-centered engagement. We will then recruit and train community action team members to develop action plans to implement the curriculum during community-located events. In the last 2 stages, we will implement and evaluate COA’s effect on PrEP awareness, interest, HIV or PrEP stigma, and connection to PrEP care among cisgender women community members.

**Results:**

This project was funded by the National Institutes of Health and approved by the Emory University institutional review board in July 2021. Data collection began in December 2021 and is ongoing. COA stage 1 of the study is complete with 70 participants enrolled. Community events commenced in November 2023, and data collection will be completed by November 2025. Stage 1 qualitative data analysis is complete with results to be published in 2024. Full study results are anticipated to be reported in 2026.

**Conclusions:**

Through a community-clinic-academic collaboration, this protocol proposes to mount a coordinated approach across diverse Atlanta counties to strengthen HIV prevention for cisgender women and to create a sustainable systems approach to move new sexual health innovations more quickly to cisgender women.

**International Registered Report Identifier (IRRID):**

DERR1-10.2196/56293

## Introduction

Initiatives for ending the HIV epidemic in the United States must include cisgender women. Among ~37,000 people diagnosed with HIV annually in the United States, 19% are cisgender women, with the majority (~60%) of new HIV diagnoses among cisgender women occurring in the South [1,2]. Georgia has the highest rate of new HIV diagnoses (21% occur among cisgender women), and also has one of the highest rates of perinatal HIV infection [1,3]. HIV diagnoses among cisgender women in Georgia and nationally are more evenly distributed across age groups than in men, with magnified disparities by race or ethnicity [2,3]. This highlights the importance of women-tailored HIV prevention care, including pre-exposure prophylaxis (PrEP) to effectively reach all who may benefit. Within Georgia, the Atlanta metropolitan area accounts for over half of all annual HIV diagnoses [3], with 4 counties (Cobb, DeKalb, Fulton, and Gwinnett) identified as a high priority in the federal Ending the HIV Epidemic (EHE) initiative [4]. Reducing HIV in Atlanta is a public health priority, and cisgender women–tailored strategies will be necessary to achieve an end to HIV.

PrEP is dramatically underused by cisgender women. There has been wide-scale endorsement to bring PrEP use to scale through dissemination and implementation efforts in the United States since its approval in 2012 [5-8]. PrEP use has since steadily increased among men but remains flat in women [9]. PrEP is underused in women relative to need, especially in the Southern United States [10]. Women are <5% of US PrEP users, and account for only 2% of the estimated 176,670 cisgender women for whom PrEP is indicated [11,12]. The first steps for increasing PrEP uptake are ensuring that those who can benefit from PrEP are aware of it, and ensuring that PrEP is accessible in settings where they seek health care [13]. Unfortunately, PrEP knowledge is low among cisgender women in the United States, including in the Southern United States) [14-20] and among women’s health care providers [21]. Encouragingly, cisgender women report interest in taking PrEP once informed [22,23], and women’s providers are willing to prescribe PrEP once trained [21]. Our group previously investigated PrEP awareness and provider PrEP knowledge in 4 Atlanta family planning clinics that were not previously providing PrEP. Clients resided in 103 (91%) of 113 metropolitan Atlanta zip codes (the majority of clients resided in zip codes with HIV prevalence >1%) [14,24]. Only 95 (19%) of 500 cisgender women clients surveyed knew about PrEP, but after a brief provider training, 72 (66%) of 110 cisgender women with PrEP indicators received PrEP counseling during their visits. After receiving PrEP counseling, clients expressed interest in PrEP if it were offered on-site rather than through off-site referral. This study demonstrated the potential reach of sexual health clinics in high HIV burden areas as PrEP delivery sites.

However, PrEP delivery in publicly funded family planning clinics in the South remains low, despite clinical guidelines incorporating PrEP [25]. We previously surveyed nearly 600 providers or staff working in 286 Title X clinics across the South in 2018; only 22% of clinics provided any PrEP services (including through referral), and the Southeastern region (including Atlanta) had the fewest clinics offering PrEP [14,26].

Once clinic capacity for PrEP is built, it does not address low PrEP awareness among cisgender women. Lessons learned from the rollout of other prevention technologies, such as human papillomavirus vaccination [27] and new contraception methods [28], highlight the need to simultaneously address demand barriers that impede use [29]. We are learning that the same applies to scaling PrEP, particularly for cisgender women in Atlanta [14,15,24,26]. To date, efforts to scale PrEP in the United States have largely focused on PrEP awareness for men who have sex with men [8], despite continued low PrEP knowledge among women [15-20]. Our 2018 research found that once informed about PrEP, cisgender women in Atlanta wanted more information and widespread awareness-building specifically for women [30]. Thus, ending the HIV epidemic among women will require addressing uptake barriers through building interest in PrEP and promoting and connecting cisgender women to women-friendly PrEP clinics.

A comprehensive systems-level approach is needed to increase PrEP reach among cisgender women. To create a proactive solution for bridging the chasm from science to practice for PrEP use among cisgender women and building on our existing clinic-academic-community partnerships, we will innovatively use the Interactive Systems Framework (ISF) [31]. The ISF is a multisystem model to guide the dissemination and implementation of prevention programming through the work of 3 interactive systems, “prevention delivery” (implements the innovation in the real world), “prevention support” (provides training and technical assistance to users in the field), and “prevention synthesis and translation” (conducts research and distills information about innovations in user-friendly formats). The ISF has been used successfully by the Centers for Disease Control and Prevention (CDC) in their multisite capacity-building effort to reduce teen pregnancies in the United States [32], showing its feasibility for building prevention infrastructure to guide large-scale prevention efforts in the area of sexual health for women [32,33]. All 3 systems, and the different stakeholders who populate each system, interact to support each other toward a common goal, which is necessary to mount successful, sustainable, large-scale adoption of new prevention innovations (eg, PrEP, including long-acting PrEP).

The ISF provides a strong model to strengthen access to PrEP care, but it lacks equally robust attention to addressing PrEP awareness and interest. Thus, in this protocol, we innovatively supplemented the ISF with an evidence-based community organizing approach (COA), previously demonstrated effective for addressing underage alcohol use [34,35]. This evidence-based approach can create broader change across the community for any community challenge [36,37]. The COA consists of 6 stages of activities. The first stage is assessing the community through one-on-one conversations with citizens, typically done by a lead community organizer (LCO). The second stage is building the base, in which an action team of approximately 15 citizens is assembled. The action team members are not typically affiliated with formal prevention programs or HIV coalitions and do not need to hold formal positions of power. Rather they are everyday citizens—a retired teacher, minister, stay-at-home parent, coach, and so forth—who care deeply about preventing HIV. The action team is then trained by the LCO on strategies for reducing HIV, including PrEP, and how to take actions to advance those evidence-based strategies (eg, where individuals can go to get these services near them). In the third stage, expanding the base, the action team builds additional rings of supporters connected to the effort. In the fourth stage, the LCO and action team members develop an action plan, wherein they schedule strategic actions designed to accomplish specific organizing outcomes (eg, sharing at community events). Stage 5 involves implementing the plan and carrying out their planned actions. Stage 6 involves assessing results, celebrating accomplishments, and refining the next steps that were planned. Thus, this COA systematically develops the leadership skills of trusted community members to become leaders and advocates for HIV prevention inclusive of PrEP for women in their communities.

We propose to innovatively use this COA to systematically develop the leadership skills and HIV prevention knowledge of individuals to become community advocates for HIV prevention in metro Atlanta to reduce HIV or PrEP-related stigma, build demand for PrEP, and facilitate linkages to women-friendly PrEP clinics. We hypothesize that the COA is feasible to implement and that the PrEP action plan implemented as part of the COA will be associated with increased PrEP awareness and interest, decreased HIV or PrEP stigma, and increased awareness about where women or individuals can get PrEP among cisgender women community members.

## Methods

### Study Setting

We will implement the COA (Textbox 1) within the 4 Atlanta high-priority EHE counties, in partnership with SisterLove, an Atlanta-based women’s HIV and reproductive justice community based organization (CBO) that is internationally recognized for raising sexual health and HIV awareness among women and has deep connections in the community, and additional CBOs identified through our formative work.

**Description of the 6-stage community organizing approach.**

**Stage 1: assess community**

Conduct one-on-one interviews to assess pre-exposure prophylaxis (PrEP) attitudes, motivations, and influences among key community members; use these findings to adapt a sexual health curriculum to address feedback.

**Stage 2: build the base**

Assemble “action teams” composed of approximately 15 individuals who work or reside across 4 metro Atlanta counties with the help of community-based organization partners and train them on an adapted sexual health curriculum.

**Stage 3: expand the base**

Identify individuals to engage during implementation (influencers to connect with to recruit women to attend workshops) and resources to contribute (eg, space to conduct the workshops).

**Stage 4: develop an action plan**

Schedule community education meetings across all 4 counties (5-10 per year per action team member) to reach diverse communities of cisgender women.

**Stage 5: implement the action plan**

Action team members conduct community events (~150 annually) using the adapted sexual health curriculum.

**Stage 6: evaluate the action plan**

Monitor the number of events, participant demographics, and fidelity to the sexual health curriculum; collect survey data to evaluate changes in PrEP awareness, interest, stigma, and reach.

### Implementation of the COA

#### Preparing for Action (COA Stages 1-4)

We will first hire and train an LCO. In collaboration with the Emory team, the LCO will assess the community (COA stage 1) through one-on-one conversations with up to 100 individuals across Atlanta; ~25 per EHE county (collaborating CBOs will assist in identifying individuals such as opinion leaders, influential partners in their work, and diverse women in the community). Interviews will capture attitudes, motivations, and influences related to women’s health and well-being, as well as their sexual health, with a focus on HIV prevention and PrEP. Interviews will be recorded and transcribed. Using an inductive coding approach, the LCO and Emory team members will code interviews and identify themes salient to address during COA implementation. We will then use findings to adapt SisterLove’s existing sexual health curriculum (Healthy Love Workshop [38]) to address community feedback salient to PrEP use among women. Healthy Love is an evidence-based single-session, a small-group intervention for women with core content designed to increase participants’ HIV and sexual health knowledge (including prevention strategies), risk perception, and self-efficacy to engage in HIV or sexually transmitted infection (STI) testing and preventative practices. The fundamental information within Healthy Love, specifically related to knowledge, risk perception, and self-efficacy for testing, will be retained, and updates will be made to incorporate information about PrEP.

Next, we will build the base (COA stage 2) by assembling action teams comprised of approximately 15 individuals who work or reside across all 4 counties with the help of CBO partners. Action teams will be essential for reaching diverse groups of women in their community and raising awareness, interest, and connection to PrEP via facilitating educational outreach. Action team members will be asked to commit to at least 1 year of engagement. They will be provided with an annual honorarium for serving in this role, and a certificate of appreciation from SisterLove and Emory teams acknowledging their significant role in reducing HIV in their communities. Action team members will work in coordination under the LCO, but each team will focus their implementation activities on their respective EHE counties. The LCO and SisterLove team will prepare the action teams with training on the adapted sexual health curriculum, supplemented with information on women-friendly places to access PrEP.

To expand the base (COA stage 3) action team members will identify individuals to engage during implementation and resources they might be able to contribute during implementation (eg, space for community education meetings to conduct adapted Healthy Love Workshop, audiences for meetings, and access to influencers to connect with to recruit women to attend meetings). As part of COA stage 4 (developing an action plan), the goal for each action team member is to arrange 5-10 community education events to be held per year in their EHE county. The LCO and action team members (supported by SisterLove and Emory teams) will schedule community education meetings reaching diverse communities of cisgender women, built on the unique reach of each action team member, across the 4 EHE counties.

#### Implementing and Evaluating the PrEP Action Plan (COA Stages 5-6)

Over 2 years, action team members will collectively conduct ~300 community events (~10 events per action team member per year) with an average of 10 community members attending each event. The action team members will facilitate their planned events (~2 hours each), following the adapted Healthy Love Workshop manual, with the assistance of the LCO, SisterLove, and the Emory team for data collection.

To monitor the implementation of the PrEP action plan, we will use electronic monitoring logs to capture (1) the number of community events scheduled or conducted by each action team member with dates and locations recorded, (2) the number and demographics of participants at each event (gathered through attendance logs at events), and (3) the number of requests for scheduling other community meetings. A study team member will collect monitoring data at each event to ensure the accuracy and completeness of logs and use a fidelity checklist to record fidelity to the adapted sexual health curriculum for each event.

We will collect data via surveys from the ~3000 community event participants to evaluate changes in PrEP awareness, interest, stigma, and reach after participating in a community education event conducted as part of the PrEP action plan. Specifically, all cisgender women participants at community events will be asked to complete brief pre- and posttest surveys that include measures on demographics, knowledge and attitudes about PrEP [15], concerns or barriers to PrEP use [39], and PrEP or HIV stigma [40,41]. Upon arriving at the event, each participant will sign in (name, email, phone number, and social media usernames) on a study laptop. Two additional questions will be asked: (1) “Is it ok to contact you in a few months to ask a few follow-up questions about the workshop—you will receive a US $5 gift card for your time?” and (2) “What is your preferred way to be contacted—text, phone, or email?” Participants will then be assigned a unique identifier to be used in the pre- and posttest surveys which will be administered electronically using a QR code link. The study team member will verify the unique identifier used with the participant ID log which will be stored separately from the participant’s contact information. Participants’ pretest surveys will be collected at the onset of the event, and posttest surveys will be collected upon completion. Study staff will review surveys upon collection and encourage participants to complete any missing items. Approximately 3 months after participation, ~50% of participants (among those agreeing to be contacted) will be randomly selected per event to participate in a brief survey (via text, email, or phone). Survey questions will be yes or no items assessing PrEP reach since participation in the community event to determine whether the participants has (1) received HIV or STI testing, (2) gone to a clinic for sexual health services, (3) talked to a health care provider about PrEP, (4) started PrEP, and (5) shared information about PrEP with other women (if so, how many). See Table 1 for a full list of evaluation measures.

**Table 1:** Comprehensive evaluation of pre-exposure prophylaxis (PrEP) action plan implementation on PrEP awareness, interest, and connection to PrEP care.

Method	Data source	Timing	Evaluation measures
Action plan implementation monitoring	Electronic monitoring log to capture the number of events, number of participants, types of PrEP resources shared, and number of requests additional events	Completed for each community event	Number of community events conducted by each action team member; number and demographics of participants at each event; and number of requests for scheduling other community meetings
Fidelity to sexual health curriculum	Fidelity checklist to record fidelity to the adapted sexual health curriculum in real time	Completed for each community event	Overall and individual checklist item
Pre- and posttest assessment of events	Brief paper-pencil pre- and posttest survey completed by ~3000 cisgender women attending community events	All cisgender women who participate in community events before and after the event	Demographics, knowledge, and interest in PrEP, PrEP stigma and attitudes about PrEP, history of PrEP use, and knowledge of PrEP clinics
Follow-up survey after events	Random sample of 50% of community event participants who agree to be contacted will be sent a brief email or text survey	Three months after participating in a community event	Yes or no questions since event: (1) received HIV testing, (2) went to Title X clinic that provides PrEP, (3) talked to a doctor about PrEP, (4) started PrEP, and (5) shared information about PrEP with other women (if so, how many)
PrEP reach among cisgender women seen in area clinics	Number of PrEP prescriptions to cisgender women seen in PrEP-providing clinics shared in community event resource list	Quarterly before, during, and after community events	Changes in number of PrEP prescriptions to women per quarter among PrEP-providing clinics

### Statistical Analysis Plan

Descriptive statistics will be conducted on monitoring and fidelity data by year for each of the 2 implementation years. Feasibility of the COA will be determined by (1) successful implementation of ~10 community events per year by each action team member (~15 members), (2) at least 100 community members reached by each action team member per year (as indicated by # of participants attending their events), and (3) high fidelity to the adapted Healthy Love by action team members per year (>80% fidelity: determined by fidelity checklist indicating >80% content completed per event).

For pre-post assessments, scores on scales will be computed according to published guidance [39-42]. Descriptive analyses and paired *t* tests or chi-square tests will be conducted to ascertain pre-post changes in PrEP awareness, interest, stigma, and knowledge of where to access PrEP. For follow-up surveys, descriptive statistics will summarize the connection between PrEP care, connection to HIV testing, and PrEP reach (ie, self-reported initiation of PrEP) among the subset of community event participants who complete a follow-up survey.

We will conduct exploratory logistic regression analyses examining the association between pre-post survey scores (PrEP knowledge, attitudes, barriers, stigma, and prior use of PrEP) on PrEP reach, including demographic factors as covariates. Finally, exploratory descriptive analysis will summarize changes in PrEP reach among cisgender women receiving care at publicly funded family planning clinics in the 4 Atlanta EHE counties (number of PrEP prescriptions to women per quarter among PrEP-providing clinics discussed or shared during community events) for each year before, during, and after PrEP action plan implementation. Given our design, it is not appropriate to conduct comparison tests for this analysis.

### Ethical Considerations

This study was approved by the Emory University institutional review board (#00002950). For the community interviews which were all completed via video conferencing, a waiver of documentation of consent was obtained, and the interview was completed after verbal consent. Community interview participants received a US $50 gift card upon completion of an interview. Community event participants who complete pre-post assessments and follow-up surveys will provide written informed consent. Community event participants will receive a US $5 gift card for completion of the 5-item 3-month follow-up text or email survey.

Interviews are conducted by trained research staff and recorded with audio recording devices; we instruct individuals to avoid using their names during the interview. In the event an identifier is used in the recording, all personally identifiable information is redacted from the transcripts during data quality assurance procedures performed by the study team. All audio recordings are professionally transcribed by a trusted vendor with secure web-based file transmission procedures. After transcription, the study team reviews each transcription against the audio recording to ensure verbatim accuracy and to remove identifiable information from the transcripts during this quality assurance procedure. After all transcripts are quality-checked and saved, the audio recordings will be destroyed. A study log kept by the study team will document this process. Transcripts and audio recordings are only stored on secure, password-protected platforms. No personal identifiers will be stored for other data collection components, except to contact community event participants for follow-up surveys and distribute participant incentives. Personal identifiers will not be linked to any other data and will be destroyed as soon as survey invitations and participant incentives have been distributed.

## Results

Study activities were funded by the National Institutes of Health (NIH) and was approved by the institutional review board in July 2021 and data collection began in December 2021 and is ongoing. From December 2021 to October 2022, we hired and trained the LCO and completed 70 community interviews with key stakeholders. Eligible participants worked or resided in Dekalb, Gwinnett, Fulton, or Cobb County in the state of Georgia and were able to provide verbal consent to participate. Participants were primarily female-identifying (n=64, 91%); of 70 total participants, 20 (29%) endorsed being a lay community member, while others reported community involvement through organizations or businesses (n=19, 27% members of a nonprofit organization; n=12, 17% staff at a clinic; n=4, 6% self-employed; n=4, 6% college students; and n=1, 1% affiliated with a faith-based organization). About 10% (n=7) of participants reported involvement in HIV support groups (n=5, 7%) or advocacy groups (n=2, 3%) as their community involvement. Of those who reported race and ethnicity (n=46, 66%), 35 (76%) identified as Black, 7 (15%) identified as White, and 4 (9%) identified as more than 1 race. A total of 4 (9%) participants reported Hispanic or Latino ethnicity which parallels HIV diagnoses by race or ethnicity in Georgia [3].

From October 2022 to December 2022, we analyzed interview data and identified important preliminary themes in these conversations including the need for broad-based, inclusive approaches to discussions about women’s sexual health and HIV prevention with accurate, straightforward, and reliable content delivered by trusted, relatable members of the community in safe and easy to access spaces. From December 2022 to June 2023, we collaborated with SisterLove on the adaptation of the Healthy Love sexual health curriculum based on these preliminary community interview findings while taking care to retain fundamental information within Healthy Love, specifically related to HIV knowledge, risk perception, and self-efficacy for testing (COA stage 1). From February 2023 to July 2023, we recruited and trained a diverse group of 15 action team members and gathered feedback on the curriculum adaptation. Additional changes to the curriculum were made based on community feedback from July to August 2023. Community events commenced in November 2023 and data collection will be completed by November 2025. Full trial results are anticipated to be reported by July 2026.

## Discussion

### Principal Findings

Using a COA that engages and supports community members in educating other community members about their sexual health can broaden and enrich the awareness, and potentially the reach of, HIV prevention, testing, and PrEP into diverse networks of cisgender women. Our findings in assessing the communities in metro Atlanta thus far have affirmed the need for greater emphasis on women-inclusive initiatives delivered by individuals considered relatable within the community who are comfortable and knowledgeable about discussing sexual health with the overarching goal of creating safe spaces for authentic conversations. Taking this into consideration when forming action teams, the LCO, with the assistance of SisterLove and Emory partners, dedicated extra time and attention during stage 1 of the COA to identify, recruit, and train team members to ensure that teams remain representative of women of varying ages, races, ethnicities, and social networks living in EHE high priority counties in metro Atlanta.

Adjustments in the protocol for the adaptation of the evidence-based Healthy Love curriculum by SisterLove, Inc were also made to include the opinions and feedback of the action team members on the revised curriculum content. Initial curriculum revisions were drafted to include content salient to data outcomes from the in-depth community interviews capturing attitudes, motivations, and influences related to women’s sexual health, with a focus on HIV prevention and PrEP. The revised draft was then presented to action team members as a community event and their feedback was gathered and later incorporated into the final curriculum to guide action teams in facilitating health education community events.

Moving forward, we anticipate that the evidenced-based COA will be feasible, and participation in its community events will be associated with increases in PrEP awareness and interest, decreases in PrEP or HIV stigma, and greater knowledge about where women can go for PrEP care (eg, PrEP-providing Title X clinics). We also expect that participation in events will be associated with improved PrEP reach among women who participate. These findings will provide critical data related to the COA use to create a potentially sustainable community-engaged approach for disseminating information about new HIV prevention products from trusted members in the community inclusive of cisgender women.

### Limitations

Action team members may discontinue participation over time. If this occurs, we will work with our collaborating CBOs and other action team members to recruit new individuals into this role. If a member withdraws before completing community events, other action team members, the LCO, and CBO staff will conduct the remaining scheduled events. We may encounter challenges related to the completion of the follow-up survey. If randomly selected individuals do not complete the survey, we will select another. We expect 90% will indicate willingness to be contacted, and we are only attempting to sample 50% of participants, so we are confident we can achieve our goal sample size.

### Conclusions

Through a community-clinic-academic collaboration and innovative application of COA, this protocol proposes to mount a coordinated approach across diverse Atlanta counties to strengthen HIV prevention for cisgender women, and importantly, to create a sustainable systems approach to move new and sexual health innovations more quickly to cisgender women.

## Abbreviations

CBO: community-based organization
CDC: Centers for Disease Control and Prevention
COA: community organizing approach
EHE: Ending the HIV Epidemic
ISF: Interactive Systems Framework
LCO: lead community organizer
NIH: National Institutes of Health
PrEP: pre-exposure prophylaxis
STI: sexually transmitted infection

## Data Availability

The data sets generated and analyzed during this study are available from the corresponding author on reasonable request upon completion of primary analyses.
