# Correction: Applying an Evidence-Based Community Organizing Approach to Strengthen HIV Prevention for Cisgender Women in US South: Protocol for a Mixed Methods Study

**DOI:** 10.2196/63249

**Published:** 2024-06-21

**Authors:** Anandi N Sheth, Dazon Dixon Diallo, Celeste Ellison, Deja L Er, Adaora Ntukogu, Kelli A Komro, Jessica M Sales

**Affiliations:** 1 Division of Infectious Diseases Department of Medicine Emory University School of Medicine Atlanta, GA United States; 2 Ponce de Leon Center Grady Health System Atlanta, GA United States; 3 SisterLove, Inc Atlanta, GA United States; 4 Department of Behavioral, Social, and Health Education Sciences Emory University Rollins School of Public Health Atlanta, GA United States; 5 Medical College of Georgia Augusta, GA United States; 6 Department of Epidemiology Emory University Rollins School of Public Health Atlanta, GA United States

In “Applying an Evidence-Based Community Organizing Approach to Strengthen HIV Prevention for Cisgender Women in US South: Protocol for a Mixed Methods Study” (JMIR Res Protoc 2024;13:e56293) an error was noted:

In the *Acknowledgments* section, the funding source number was incorrectly listed as R01MH121962. It has been revised to:

This study is funded by the National Institutes of Health (NIH; R01MH128045).

The correction will appear in the online version of the paper on the JMIR Publications website on June 21, 2024, together with the publication of this correction notice. Because this was made after submission to PubMed, PubMed Central, and other full-text repositories, the corrected article has also been resubmitted to those repositories.

